# Efficacy of Oral Cryotherapy on Oral Mucositis Prevention in Patients with Hematological Malignancies Undergoing Hematopoietic Stem Cell Transplantation: A Meta-Analysis of Randomized Controlled Trials

**DOI:** 10.1371/journal.pone.0128763

**Published:** 2015-05-29

**Authors:** Li Wang, Zhenyang Gu, Ruiren Zhai, Shasha Zhao, Lan Luo, Dandan Li, Xiaoli Zhao, Huaping Wei, Zhaoxia Pang, Lili Wang, Daihong Liu, Quanshun Wang, Chunji Gao

**Affiliations:** 1 Department of Hematology, Chinese People’s Liberation Army (PLA) General Hospital, Beijing, China; 2 Department of Hematology and Oncology, Laoshan Branch, Chinese PLA 401 Hospital, Qingdao, China; Cardiff University, UNITED KINGDOM

## Abstract

**Objectives:**

Controversy exists regarding whether oral cryotherapy can prevent oral mucositis (OM) in patients with hematological malignancies undergoing hematopoietic stem cell transplantation (HSCT). The aim of the present meta-analysis was to evaluate the efficacy of oral cryotherapy for OM prevention in patients with hematological malignancies undergoing HSCT.

**Methods:**

PubMed and the Cochrane Library were searched through October 2014. Randomized controlled trials (RCTs) comparing the effect of oral cryotherapy with no treatment or with other interventions for OM in patients undergoing HSCT were included. The primary outcomes were the incidence, severity, and duration of OM. The secondary outcomes included length of analgesic use, total parenteral nutrition (TPN) use, and length of hospital stay.

**Results:**

Seven RCTs involving eight articles analyzing 458 patients were included. Oral cryotherapy significantly decreased the incidence of severe OM (RR = 0.52, 95% CI = 0.27 to 0.99) and OM severity (SMD = -2.07, 95% CI = -3.90 to -0.25). In addition, the duration of TPN use and the length of hospitalization were markedly reduced (SMD = -0.56, 95% CI = -0.92 to -0.19; SMD = -0.44, 95% CI = -0.76 to -0.13; respectively). However, the pooled results were uncertain for the duration of OM and analgesic use (SMD = -0.13, 95% CI = -0.41 to 0.15; SMD = -1.15, 95% CI = -2.57 to 0.27; respectively).

**Conclusions:**

Oral cryotherapy is a readily applicable and cost-effective prophylaxis for OM in patients undergoing HSCT.

## Introduction

Hematopoietic stem cell transplantation (HSCT) is a curative treatment for most hematological malignancies [1, 2]. Oral mucositis (OM), which is characterized by inflammatory and ulcerative reactions in the oral cavity [3], often results from the cytotoxic effects of chemotherapy on the epithelial cells of the oral mucosa [4]. OM is a severe and debilitating complication that is frequently encountered after HSCT. It occurs in approximately 80% of patients who receive high-dose chemotherapy as conditioning for HSCT [5], particularly with conditioning regimens containing high-dose melphalan. In addition, graft-versus-host disease (GVHD) prophylaxis that includes methotrexate (MTX) has also been associated with an increased incidence of OM [2, 6]. OM has been associated with malnutrition, the need for total parenteral nutrition (TPN), analgesic use, high risk of infection, and prolonged hospitalization [4, 7, 8]. As a result, OM dramatically impairs the quality of life of patients and increases hospital costs [2, 4].

Currently, various strategies and agents have been described for the prevention of OM, including routine oral care, mucosal surface protectants, anti-inflammatory drugs, growth factors, certain antimicrobial formulations, laser therapy, oral cryotherapy, and specific natural and miscellaneous agents. These approaches encompass a diversity of mechanisms, but the results have been controversial, and the optimal prophylaxis remains unknown [2, 4, 5, 9].

Oral cryotherapy, which is the application of ice chips to the buccal mucosa during the administration of chemotherapeutic agents, has been used to manage OM in a number of clinical trials [4, 10–14]. This treatment causes local vasoconstriction and decreased blood flow to the oral mucosa, resulting in decreased exposure of the oral mucosa to cytotoxic drugs [3, 15]. In contrast to other strategies and agents, oral cryotherapy is a readily applicable and cost-effective method in clinical settings. Limited evidence has suggested that oral cryotherapy prevents OM in patients receiving chemotherapy, particularly 5-fluorouracil or high-dose melphalan-based conditioning regimens [2, 9, 16]. However, whether oral cryotherapy can prevent OM in patients with hematological malignancies undergoing HSCT has been controversial. The results of relevant randomized controlled trials (RCTs) have varied greatly, and the sample sizes have been small.

Thus, we conducted this meta-analysis based on the data from seven RCTs. We addressed whether oral cryotherapy had clinical benefit for patients with hematological malignancies undergoing HSCT.

## Methods

### Search Strategy

We systematically conducted a literature search for RCTs evaluating the efficacy of oral cryotherapy for OM in patients undergoing HSCT. We searched PubMed and the Cochrane Library through October 2014, combining the following search terms: ‘cryotherapy’, ‘oral cooling’, ‘mucositis’, and ‘stomatitis’. The search criteria are listed in S1 and S2 Tables. The language was restricted to English. In addition, the references within the identified reports were manually searched.

### Selection Criteria

RCTs evaluating the efficacy of oral cryotherapy versus placebo, no treatment, or other interventions in patients with hematological malignancies undergoing HSCT were included in our meta-analysis, irrespective of the characteristics of the patients, the conditioning regimens, or the types of transplantation. The primary outcomes included the incidence, severity, and duration of OM. The severity of OM was assessed using the World Health Organization (WHO) grading scale, the National Cancer Institute (NCI) Common Toxicity Criteria, or the Oral Mucositis Assessment Score (OMAS). Secondary outcomes included the duration of analgesic use, the duration of TPN use, and length of hospital stay.

### Data Extraction

Two researchers extracted the data independently. All of the extracted data included the following: the study’s first author, year of publication, country of origin, period of enrollment, and sample size; the patients’ ages; oral cryotherapy protocols and non-cryotherapy protocols; and results regarding the outcomes. The discrepancies were resolved by discussion or by resorting to a specialist. We contacted the corresponding author to obtain complete data when necessary.

### Methodological Quality Evaluation

Two researchers independently assessed the methodological quality of each included study according to the Cochrane Collaboration Reviewers’ Handbook, and they assigned values of ‘high’, ‘low’, or ‘unclear’ to the following items: randomization, allocation concealment, blinding, incomplete outcome data, selective reporting, and other biases. Trials with one or more items assigned as ‘high’ were considered to be at high risk for bias. Trials with all items assigned as ‘low’ were considered to be at low risk for bias. Other trials were considered to be at unclear risk for bias [17].

### Statistical Methods

The risk ratio (RR), together with the 95% confidence interval (CI), was used for pooled dichotomous outcomes, and the standardized mean difference (SMD), together with the 95% CI, was used for continuous outcomes. Missing standard deviations were estimated using the methods in the Cochrane Handbook for Systematic Reviews of Interventions (5.0.2). Heterogeneity was assessed using the I^2^ statistic. An I^2^ >50% and a p value less than or equal to 0.10 indicated significant heterogeneity. Next, a subgroup meta-analysis or a sensitivity analysis was conducted to explain the source of the heterogeneity, if possible. A random effects model was used to conduct the meta-analysis, irrespective of whether heterogeneity existed or not. Publication bias was assessed by constructing a funnel plot and was confirmed with Egger’s test if at least 10 studies were included. The figure of assessment for the risk of bias was obtained using the Review Manager 5.1 software (The Nordic Cochrane Centre, Copenhagen, Denmark). Statistical analyses were performed with the Stata 12.0 software (Stata Corporation, College Station, TX, USA).

## Results

### Study Selection and Characteristics

**Fig 1:**
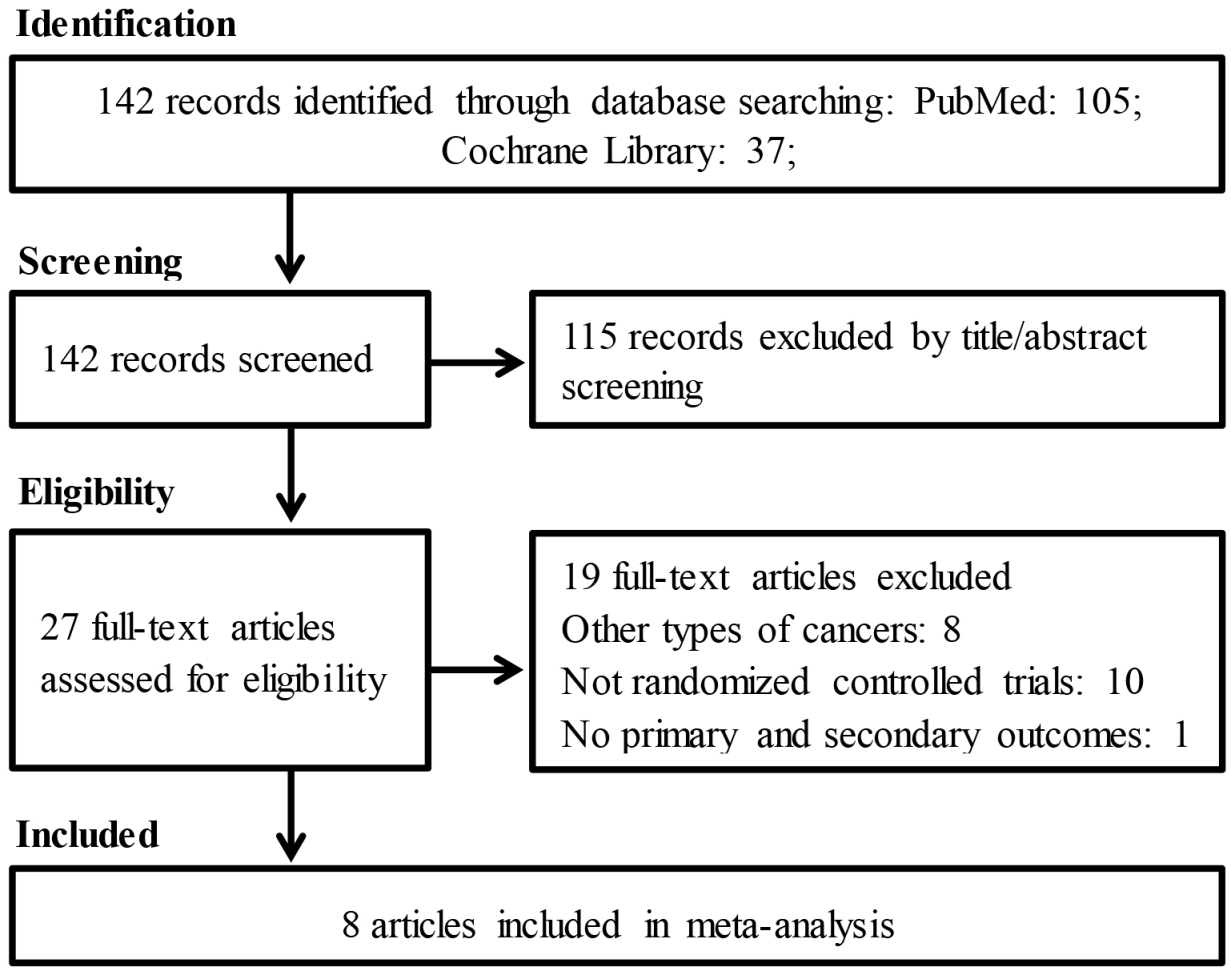
Flowchart of study selection.

A total of 142 potentially relevant records were identified through database and manual searching, as delineated in Fig 1. After screening the titles and abstracts, 115 non-relevant studies were excluded. The full texts of the remaining 27 studies were assessed, and 19 studies were discarded because they did not meet our eligibility criteria. The excluded full-text studies, with the reasons for exclusion, are listed in S3 Table. It is important to note that Svanberg et al. conducted one RCT and published two articles [18, 19]. Generally, only the most recent publication should be included from duplicate reports identified from the same trial. However, there were no duplicate data in these two articles, which reported entirely different outcomes. The first article reported the severity of OM and the length of analgesic use, while the second article reported the incidence of OM, the length of TPN use, and the length of hospital stay. All of these outcomes were included in our pooled meta-analysis; therefore, both articles were included in our meta-analysis. Ultimately, seven RCTs involving eight articles were included in our meta-analysis. The characteristics of the included studies are listed in Tables 1 and 2. Two RCTs were conducted in the USA, one in Sweden, one in Italy, one in Canada, one in Brazil, and one in China. The sample sizes of the included studies ranged from 24 to 122. One study compared oral cryotherapy to routine oral care [18] (supplemented by another article [19]), two studies compared oral cryotherapy to a normal saline rinse [20, 21], two studies compared oral cryotherapy plus oral care to oral care alone [22, 23], one study compared oral cryotherapy to no treatment [24], and one study compared oral cryotherapy plus laser therapy to laser therapy alone [25]. One study evaluated the role of cryotherapy in the prevention of MTX-based GVHD prophylaxis-induced OM [24]. One study evaluated the role of oral cryotherapy in patients undergoing busulfan/cyclophosphamide (BU/CY)-based myeloablative conditioning and HSCT [23]. The other studies evaluated the role of oral cryotherapy in patients undergoing high-dose melphalan-based conditioning followed by HSCT [18–22, 25]. One study prospectively compared oral cryotherapy plus laser therapy to laser therapy alone; this study also included a retrospective control group, and we only extracted the data from the prospective part of the study [25]. Two studies were reported as abstracts [21, 23].

**Table 1:** Characteristics of included RCTs comparing cryotherapy versus no treatment or other interventions (1).

Author	Patients	Type of HSCT, conditioning regimens	Cryotherapy group	Non-cryotherapy group
Svanberg [18, 19]	Patients with MM, lymphoma, AML, ALL, and others	79.5% of patients underwent auto-HSCT, and 20.5% underwent allo-HSCT. The majority received high-dose melphalan or BEAC conditioning.	Oral cryotherapy (sucking on ice chips or rinsing with ice cold water) started concurrently with the chemotherapy session and lasted until the end of the chemotherapy session.	Routine oral care
Lilleby [20]	Patients with MM	Auto-HSCT with conditioning containing high-dose melphalan	Oral cryotherapy (placing ice chips in the mouth) started 30 minutes before and continued for 6 hours after high-dose melphalan.	Room temperature normal saline rinse
Toro [21]	Patients with MM	Auto-HSCT with conditioning containing high-dose melphalan	Oral cryotherapy plus saline solution	Saline solution
Salvador [22]	Patients with MM	Auto-HSCT with conditioning containing high-dose melphalan	Oral cryotherapy (sucking on ice chips) started 5 minutes before, during, and after the administration of melphalan for a total of 60 minutes, plus oral care.	Routine oral care
Lu [23]	Patients with AML, ALL, and others	The majority of patients underwent allo-HSCT after conditioning with BU/CY.	Oral cryotherapy started from the beginning of drug infusion continuously until the end of drug infusion, plus oral care.	Routine oral care
Gori [24]	Patients with AML, ALL, CML, CLL, AA, MDS, HL, NHL, MM, and others	Myeloablative allo-HSCT, MTX was given as graft-versus-host disease prophylaxis.	Oral cryotherapy (applied ice chips or popsicles) started at the time when MTX was administered and was maintained for at least 1 hour.	No treatment
de Paula Eduardo [25]	Patients with MM, HL, NHL, and others	The majority of patients underwent auto-HSCT after conditioning with high-dose melphalan or BEAM, except that 7.4% in the cryotherapy plus laser therapy group underwent allo-HSCT.	Oral cryotherapy (maintained ice chips inside mouth) started 5 minutes before and continued during melphalan infusion for an additional 30 minutes after melphalan administration, followed by laser therapy.	Laser therapy

Abbreviations: RCTs: randomized controlled trials; MTX: methotrexate; ALL: acute lymphoblastic leukemia; AML: acute myeloid leukemia; CML: chronic myelogenous leukemia; CLL: chronic lymphocytic leukemia; MDS: myelodysplastic syndrome; MM: multiple myeloma; AA: aplastic anemia; HL: Hodgkin’s lymphoma; NHL: non-Hodgkin’s lymphoma; allo-HSCT: allogeneic hematopoietic stem cell transplantation; auto-HSCT: autologous hematopoietic stem cell transplantation; BEAC: conditioning regimen including carmustine, etoposide, cytarabine, and cyclophosphamide; BEAM: conditioning regimen including carmustine, etoposide, cytarabine, and melphalan; BU/CY: busulfan/cyclophosphamide.

**Table 2:** Characteristics of included RCTs comparing cryotherapy versus no treatment or other interventions (2).

Author	Publication year	Country	Enrollment period	Sample size	Age (years)	Cryotherapy benefit (study conclusion)
Svanberg [18, 19]	2007/2010	Sweden	2002–2004	78	49.8 (14.4); 54.3 (11.0)	Severity of OM: yes (auto-HSCT); Duration of analgesic use: yes; Need for TPN: yes; Length of hospital stay: yes (allo-HSCT)
Lilleby [20]	2006	USA	2003–2005	40	59 (51–71); 57 (33–72)	Severity of OM: yes; Incidence of OM: yes; Duration of OM: yes; OM related pain: yes; Duration of analgesic use: yes; Duration of TPN: yes; Length of hospital stay: no
Toro [21]	2014	USA	Not reported*	78	62 (39–75); 61.5 (43–70)	Severity of OM: yes; Incidence of OM: yes; Duration of OM: yes; Need for analgesia: yes
Salvador [22]	2012	Canada	2007	45	56.0 (8.9); 62.0 (7.7)	Severity of OM: yes; OM related pain: yes; Need for analgesia: yes; Length of hospital stay: no
Lu [23]	2013	China	Not reported*	24	35.67 (NA); 32.5(NA)	Incidence of severe OM: no; Duration of severe OM: no
Gori [24]	2007	Italy	2004–2006	122	35.5 (9–59); 40 (8–66)	Severity of OM: no; Incidence of OM: no; Duration of OM: no
de Paula Eduardo [25]	2014	Brazil	2009–2011	71	57 (6–73); 62 (43–72)	Severity of OM: yes; Duration of OM: yes

Abbreviations: RCTs: randomized controlled trials; OM: oral mucositis; TPN: total parenteral nutrition; allo-HSCT: allogeneic hematopoietic stem cell transplantation; auto-HSCT: autologous hematopoietic stem cell transplantation;

*: presented as abstract;

NA: not available.

### Risk of Bias

Random sequence generation was mentioned in all of the studies, but only one study provided an adequate description [22]. Allocation concealment was only conducted adequately in one study [22]. Blinding of the participants was impossible due to the substantial differences between oral cryotherapy and the other interventions, while only one study reported blinding to outcome assessment [22]. Ultimately, all of the studies were considered to be at high risk for bias (S1 Fig).

### Incidence, Severity and Duration of OM

**Fig 2:**
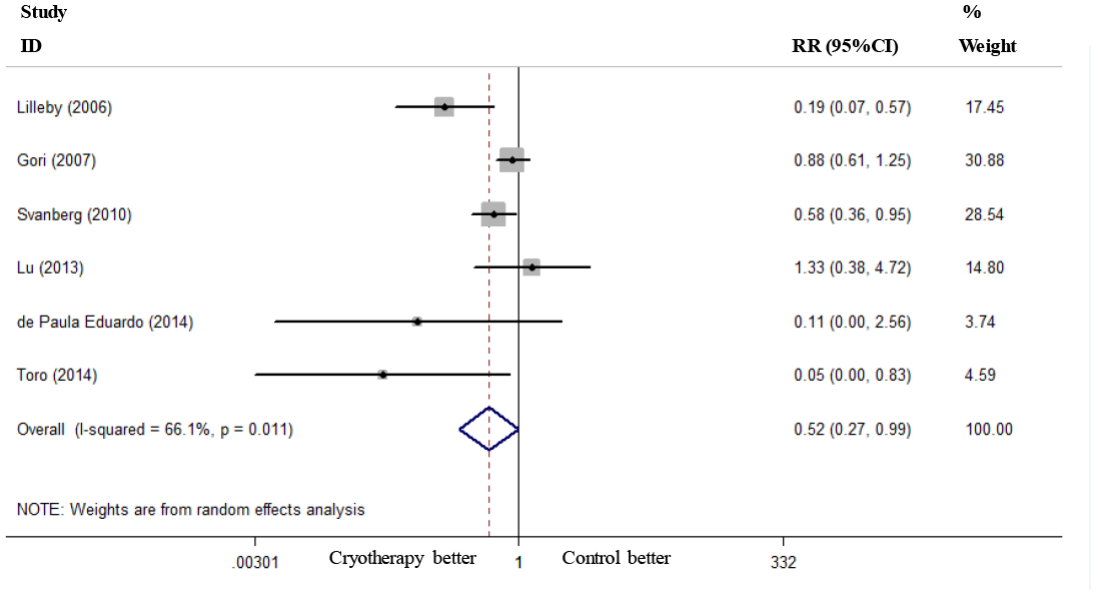
Effect of oral cryotherapy on the incidence of oral mucositis in patients with hematological malignancies undergoing hematopoietic stem cell transplantation. RR: risk ratio; CI: confidence interval.

**Fig 3:**
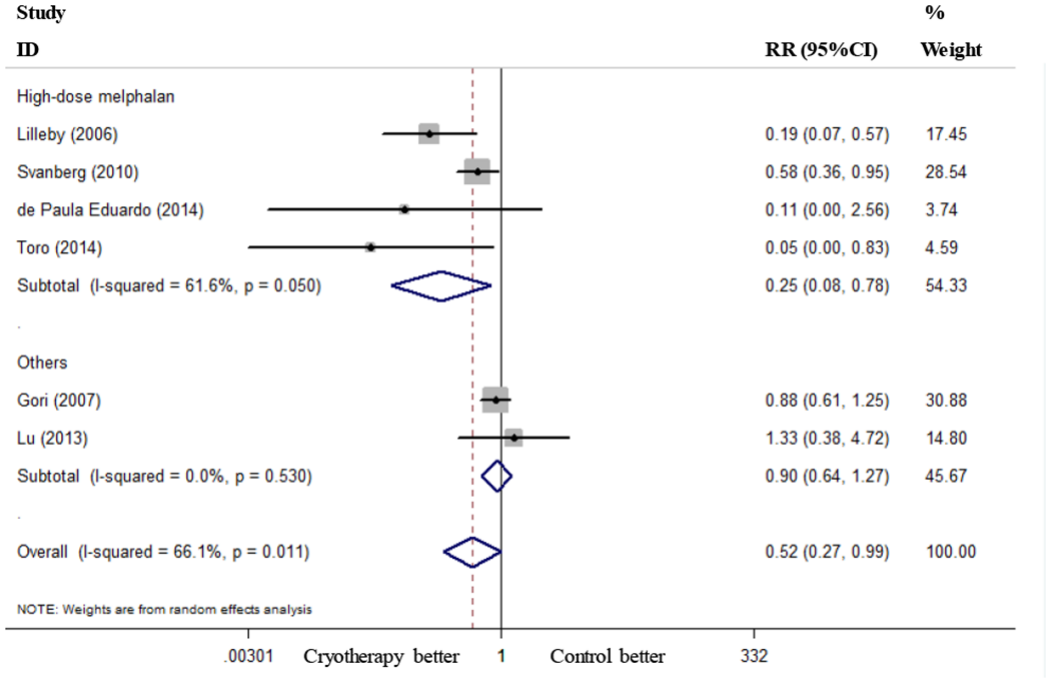
Subgroup analysis of the effects of oral cryotherapy on the incidence of oral mucositis in patients with hematological malignancies undergoing hematopoietic stem cell transplantation. RR: risk ratio; CI: confidence interval.

**Fig 4:**
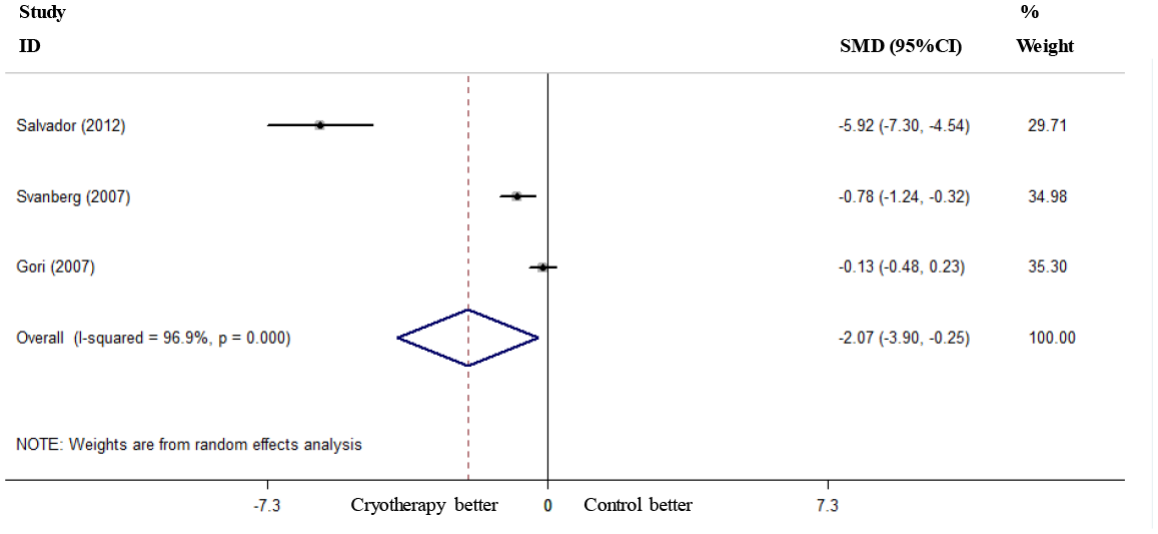
Effect of oral cryotherapy on the severity of oral mucositis in patients with hematological malignancies undergoing hematopoietic stem cell transplantation. SMD: standardized mean difference; CI: confidence interval.

**Fig 5:**
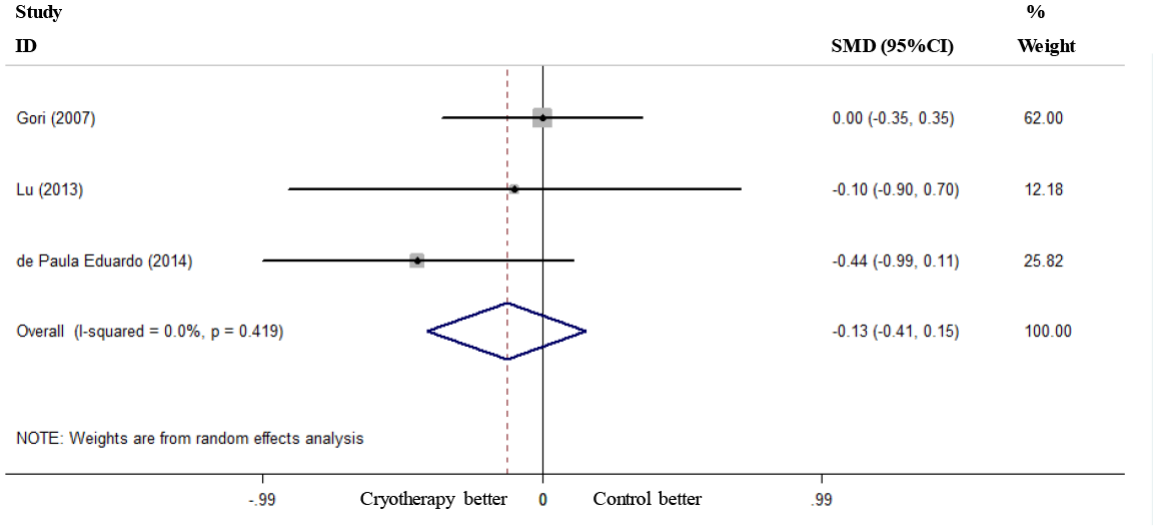
Effect of oral cryotherapy on the duration of oral mucositis in patients with hematological malignancies undergoing hematopoietic stem cell transplantation. SMD: standardized mean difference; CI: confidence interval.

Six RCTs reported the incidence of severe OM (grades 3–4) [19–21, 23–25], and these RCTs were included in the present meta-analysis. Oral cryotherapy significantly decreased the incidence of severe OM (RR = 0.52, 95% CI = 0.27 to 0.99; I^2^ = 66.1%, p = 0.011; Fig 2). We conducted a subgroup meta-analysis based on different conditioning regimens. Oral cryotherapy was associated with a low incidence of severe OM for patients who received high-dose melphalan-based conditioning (RR = 0.25, 95% CI = 0.08 to 0.78). However, the results were uncertain for other causes of induced OM (RR = 0.90, 95% CI = 0.64 to 1.27; Fig 3). Svanberg et al.’s study may have resulted in significant heterogeneity (I^2^ = 61.6%, p = 0.050). When we excluded this study and performed a meta-analysis of the remaining three studies [20, 21, 25], the heterogeneity decreased; oral cryotherapy was similarly associated with a low incidence of severe OM (RR = 0.16, 95% CI = 0.06 to 0.41; S2 Fig), which confirmed the preventive effect of oral cryotherapy on high-dose melphalan-induced severe OM. Three RCTs reported the severity of OM (maximum mucositis score) [18, 22, 24] and used pooled data. The results showed that oral cryotherapy significantly decreased OM severity (SMD = -2.07, 95% CI = -3.90 to -0.25; Fig 4). Three RCTs reported the duration of OM [23–25], and the results of the meta-analysis showed that there was no significant difference between the oral cryotherapy and non-cryotherapy groups with regard to the duration of OM (SMD = -0.13, 95% CI = -0.41 to 0.15; Fig 5).

### Length of Analgesic Use, TPN Use and Hospital Stay

**Fig 6:**
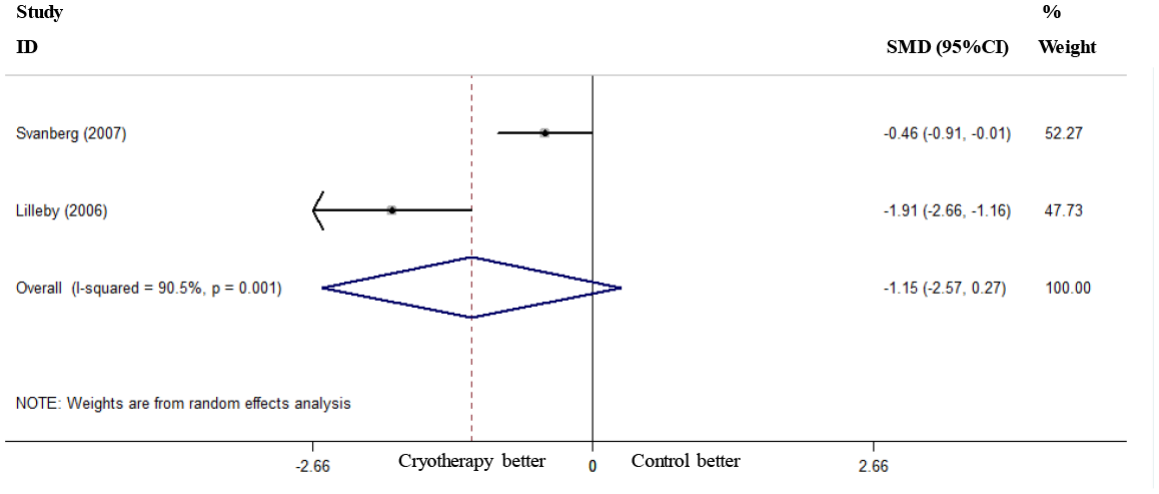
Effect of oral cryotherapy on the duration of analgesic use in patients with hematological malignancies undergoing hematopoietic stem cell transplantation. SMD: standardized mean difference; CI: confidence interval.

**Fig 7:**
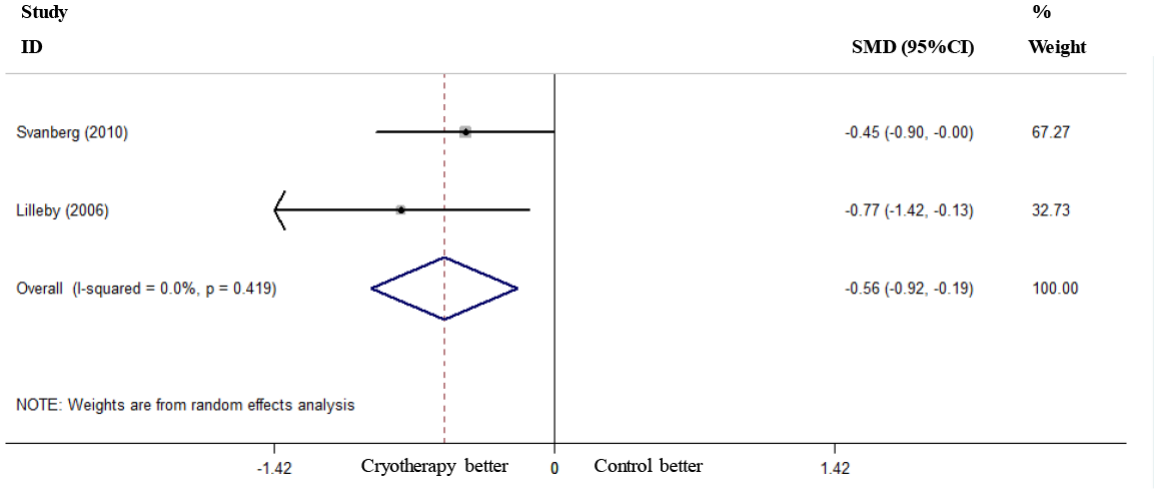
Effect of oral cryotherapy on the duration of total parenteral nutrition use in patients with hematological malignancies undergoing hematopoietic stem cell transplantation. SMD: standardized mean difference; CI: confidence interval.

**Fig 8:**
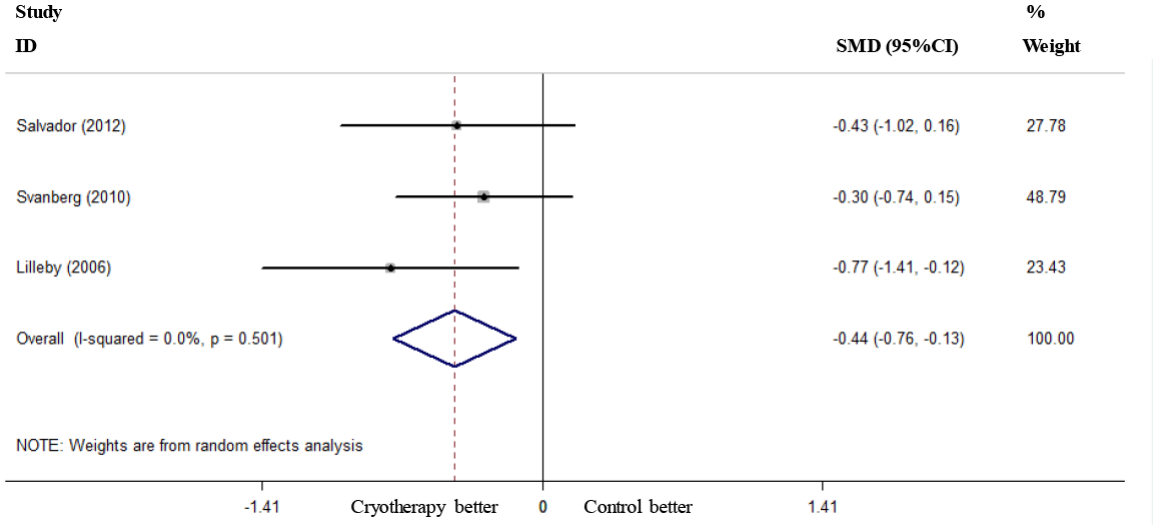
Effect of oral cryotherapy on the length of hospital stay in patients with hematological malignancies undergoing hematopoietic stem cell transplantation. SMD: standardized mean difference; CI: confidence interval.

Two RCTs reported the length of analgesic use [18, 20], and these studies were used to pool the data. The results showed that oral cryotherapy did not decrease the length of analgesic use (SMD = -1.15, 95% CI = -2.57 to 0.27; Fig 6). Two RCTs reported the length of TPN use [19, 20]; the results of the meta-analysis showed that oral cryotherapy significantly shortened TPN use (SMD = -0.56, 95% CI = -0.92 to -0.19; Fig 7). Three RCTs reported the length of hospital stay [19, 20, 22]; the results of the meta-analysis showed that oral cryotherapy significantly decreased the length of hospital stay (SMD = -0.44, 95% CI = -0.76 to -0.13; Fig 8).

## Discussion

To our knowledge, this meta-analysis was the first to focus on evaluating the efficacy of oral cryotherapy for OM in patients with hematological malignancies undergoing HSCT. OM brings great discomfort to patients, is typically painful, influences nutritional intake, and dramatically impairs patients’ quality of life. In addition, OM results in a considerable burden to the health care system, because it results in more costs associated with nutritional support, pharmacological pain management and hospitalization [5].

Our meta-analysis showed that oral cryotherapy significantly decreased the incidence and severity of OM, consistent with the guidelines of the Multinational Association of Supportive Care in Cancer and the International Society of Oral Oncology (MASCC/ISOO) [5, 26]. The subgroup and sensitivity analysis confirmed that oral cryotherapy prevented high-dose melphalan-induced OM. Svanberg et al.’s study accounted for a significant fraction of the heterogeneity. The reason for this heterogeneity might be that the author reported and analyzed the data on autologous HSCT and allogeneic HSCT separately. However, the results were uncertain for other causes of induced OM. Our meta-analysis did not show that oral cryotherapy significantly decreased the duration of OM. However, it is noted that only three studies included this outcome, and there was a trend toward a reduction in the duration of OM.

In addition, our meta-analysis showed that oral cryotherapy significantly decreased the duration of TPN use and length of hospitalization. With regard to the length of analgesic use, although there was no significant difference between the cryotherapy and non-cryotherapy groups, it is important to mention that there was a trend toward a reduction in the length of analgesic use.

This meta-analysis had several limitations that should be considered. First, there were only seven RCTs that were included in our meta-analysis, and the sample sizes of most of the included studies were small. Second, the methodological quality of all of the included studies was low due to the infeasibility of utilizing a double-blind study design, which might have resulted in bias. Third, although this meta-analysis showed that oral cryotherapy was effective in patients undergoing HSCT, it should be noted that most of the included studies involved high-dose melphalan-based conditioning regimens. Oral cryotherapy is thought to be effective only for chemotherapeutic agents with short plasma half-lives [14], and we could not draw conclusions regarding its efficacy in other conditioning regimens.

In summary, oral cryotherapy provides readily applicable, cost-effective prophylaxis for OM for patients undergoing HSCT. However, the results of this analysis must be interpreted with caution due to the small sample size, high heterogeneity and risk of bias. Future adequately powered RCTs are required.

## Supporting Information

S1 Fig Risk of bias. (TIF)

S2 Fig Sensitivity analysis of the effect of oral cryotherapy on the incidence of oral mucositis. RR: risk ratio; CI: confidence interval.
(TIF)

S1 PRISMA Checklist PRISMA Checklist. (PDF)

S1 Table Search criteria for PubMed (from inception to Oct. 31, 2014). (PDF)

S2 Table Search criteria for the Cochrane Library (from inception to Oct. 31, 2014). (PDF)

S3 Table Characteristics of excluded full-text studies. (PDF)
