# Supplementary figures and images for: Efficacy of Oral Cryotherapy on Oral Mucositis Prevention in Patients with Hematological Malignancies Undergoing Hematopoietic Stem Cell Transplantation: A Meta-Analysis of Randomized Controlled Trials
Source: PLoS One. 2015 May 29;10(5):e0128763. doi: 10.1371/journal.pone.0128763 (PMC4449217; doi:10.1371/journal.pone.0128763)

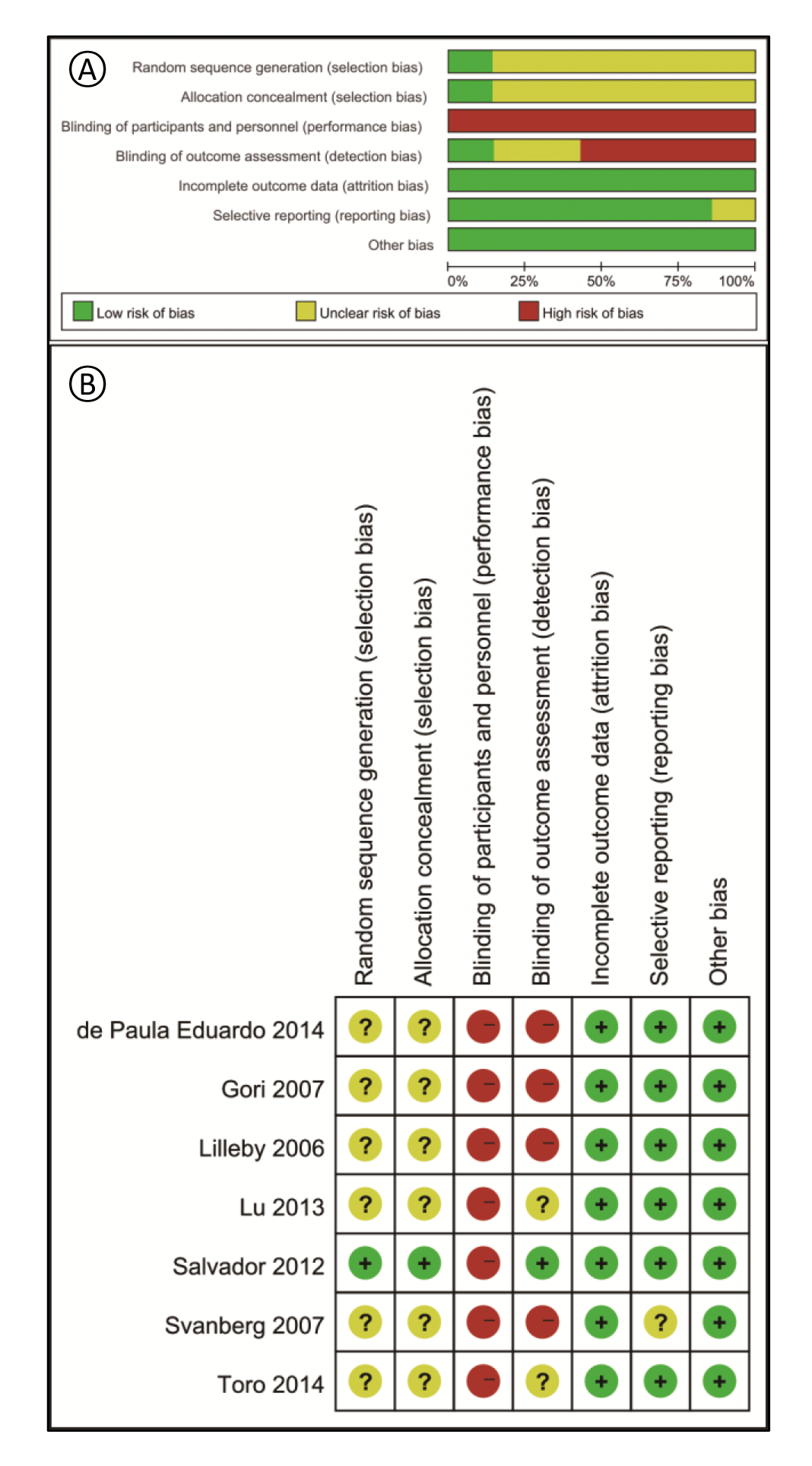

Supplement: S1 Fig — (TIF) [file pone.0128763.s001.tif]

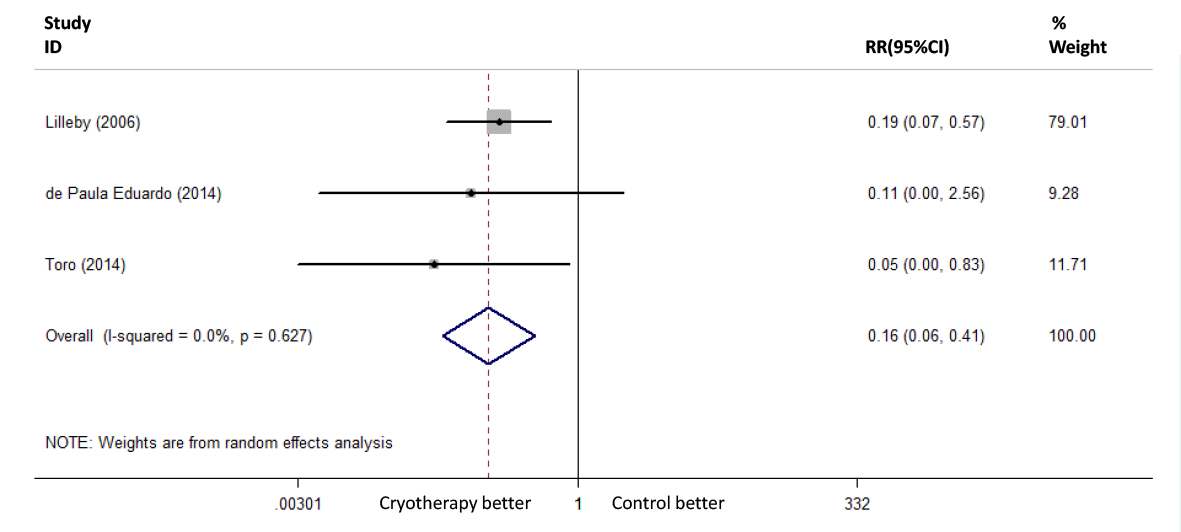

Supplement: S2 Fig — RR: risk ratio; CI: confidence interval. (TIF) [file pone.0128763.s002.tif]
