# Supplementary material for: Efficacy of Oral Cryotherapy on Oral Mucositis Prevention in Patients with Hematological Malignancies Undergoing Hematopoietic Stem Cell Transplantation: A Meta-Analysis of Randomized Controlled Trials
Source: PLoS One. 2015 May 29;10(5):e0128763. doi: 10.1371/journal.pone.0128763 (PMC4449217; doi:10.1371/journal.pone.0128763)
Supplement: S1 Table — (PDF) [file pone.0128763.s004.pdf]

Table S1 Search criterion of PubMed (from inception to Oct 31, 2014)

No.	Query Results	Results
#10	Search ((((oral cooling) OR cryotherapy) OR "Cryotherapy"[Mesh])) AND ((((mucositis) OR stomatitis) OR "Stomatitis"[Mesh]) OR ("Mucositis"[Mesh] OR "Stomatitis"[Mesh]))	105
#9	Search (((mucositis) OR stomatitis) OR "Stomatitis"[Mesh]) OR ("Mucositis"[Mesh] OR "Stomatitis"[Mesh])	29941
#8	Search "Mucositis"[Mesh] OR "Stomatitis"[Mesh]	14162
#7	Search "Stomatitis"[Mesh]	13458
#6	Search stomatitis	24667
#5	Search mucositis	6892
#4	Search ((oral cooling) OR cryotherapy) OR "Cryotherapy"[Mesh]	24938
#3	Search "Cryotherapy"[Mesh]	20300
#2	Search cryotherapy	24294
#1	Search oral cooling	694
