# Supplementary material for: Efficacy of Oral Cryotherapy on Oral Mucositis Prevention in Patients with Hematological Malignancies Undergoing Hematopoietic Stem Cell Transplantation: A Meta-Analysis of Randomized Controlled Trials
Source: PLoS One. 2015 May 29;10(5):e0128763. doi: 10.1371/journal.pone.0128763 (PMC4449217; doi:10.1371/journal.pone.0128763)
Supplement: S2 Table — (PDF) [file pone.0128763.s005.pdf]

Table S2 Search criterion of Cochrane Library (from inception to Oct 31, 2014)

No.	Query Results	Results
#1	oral cooling:ti,ab,kw (Word variations have been searched)	99
#2	cryotherapy:ti,ab,kw (Word variations have been searched)	946
#3	MeSH descriptor: [Cryotherapy] explode all trees	1197
#4	#1 or #2 or #3	1752
#5	mucositis:ti,ab,kw (Word variations have been searched)	1397
#6	stomatitis:ti,ab,kw (Word variations have been searched)	1653
#7	MeSH descriptor: [Mucositis] explode all trees	73
#8	MeSH descriptor: [Stomatitis] explode all trees	693
#9	#5 or #6 or #7 or #8	2725
#10	#4 and #9	37
