# Supplementary material for: Efficacy of Oral Cryotherapy on Oral Mucositis Prevention in Patients with Hematological Malignancies Undergoing Hematopoietic Stem Cell Transplantation: A Meta-Analysis of Randomized Controlled Trials
Source: PLoS One. 2015 May 29;10(5):e0128763. doi: 10.1371/journal.pone.0128763 (PMC4449217; doi:10.1371/journal.pone.0128763)
Supplement: S3 Table — (PDF) [file pone.0128763.s006.pdf]

Table S3 Characteristics of excluded full-text studies

Studies	Reasons for exclusion
Mahood 1991 [1]	Cancer type unclear, 5-fluorouracil related stomatitis
Rocke 1993 [2]	Cancer type unclear, 5-fluorouracil related stomatitis
Cascinu 1994 [3]	Colon-rectum, pancreas, stomach, prostate cancers, 5-fluorouracil induced stomatitis
Nikoletti 2005 [4]	Colorectal cancer, 5-fluorouracil related stomatitis, plain ice versus flavoured ice,
Karagozoglu 2005 [5]	Epidermoid cancer, small cell cancer, adenocarcinoma, mesothelioma
Baydar 2005 [6]	Carcinomas of the colon, rectum, stomach, head of pancreas, cecum, sigmoid, neuroendocrine tumors, and metastatic tumors of unknown origin of the liver.
Papadeas 2007 [7]	Cancer type unclear
Katranci 2012 [8]	Gastric cancer, colon cancer, pancreatic cancer, and rectal cancer
Dumontet 1994 [9]	Case series
Aisa 2005 [10]	Historical control
Mori 2006 [11]	Historical control, oral cryotherapy 60 minutes versus 120 minutes
Sato 2006 [12]	No randomization, combination of cryotherapy with propantheline versus cryotherapy or propantheline alone, or no treatment.
Ohbayashi 2008 [13]	Not RCTs
Bhatt 2010 [14]	Historical control
Vokurka 2011 [15]	Observational study
Vokurka 2012 [16]	Not RCTs
Vokurka 2014 [17]	Not RCTs
Batlle 2014 [18]	Retrospective cohort study
Svanberg 2012 [19]	No primary and secondary outcomes

Note: RCTs: randomized controlled trials

References

1 Mahood DJ, Dose AM, Loprinzi CL, Veeder MH, Athmann LM, Therneau TM *et al.* Inhibition of fluorouracil-induced stomatitis by oral cryotherapy. J Clin Oncol, 1991;9(3):449-452.

2 Rocke LK, Loprinzi CL, Lee JK, Kunselman SJ, Iverson RK, Finck G *et al.* A randomized clinical trial of two different durations of oral cryotherapy for prevention of 5-fluorouracil-related stomatitis. Cancer, 1993;72(7):2234-2238.

3 Cascinu S, Fedeli A, Fedeli SL, Catalano G. Oral cooling (cryotherapy), an effective treatment for the prevention of 5-fluorouracil-induced stomatitis. Eur J Cancer B Oral Oncol, 1994;30b(4):234-236.

4 Nikoletti S, Hyde S, Shaw T, Myers H, Kristjanson LJ. Comparison of plain ice and flavoured ice for preventing oral mucositis associated with the use of 5 fluorouracil. J Clin Nurs, 2005;14(6):750-753.

5 Karagozoglu S, Filiz Ulusoy M. Chemotherapy: the effect of oral cryotherapy on the development of mucositis. J Clin Nurs, 2005;14(6):754-765.

6 Baydar M, Dikilitas M, Sevinc A, Aydogdu I. Prevention of oral mucositis due to 5-fluorouracil treatment with oral cryotherapy. J Natl Med Assoc, 2005;97(8):1161-1164.

7 Papadeas E, Naxakis S, Riga M, Kalofonos C. Prevention of 5-fluorouracil-related stomatitis by oral cryotherapy: a randomized controlled study. Eur J Oncol Nurs, 2007;11(1):60-65.

8 Katranci N, Ovayolu N, Ovayolu O, Sevinc A. Evaluation of the effect of cryotherapy in preventing oral mucositis

associated with chemotherapy - a randomized controlled trial. Eur J Oncol Nurs, 2012;16(4):339-344.

9 Dumontet C, Sonnet A, Bastion Y, Salles G, Espinouse D, Coiffier B. Prevention of high dose L-PAM-induced mucositis by cryotherapy. Bone Marrow Transplant, 1994;14(3):492-494.

10 Aisa Y, Mori T, Kudo M, Yashima T, Kondo S, Yokoyama A *et al.* Oral cryotherapy for the prevention of high-dose melphalan-induced stomatitis in allogeneic hematopoietic stem cell transplant recipients. Support Care Cancer, 2005;13(4):266-269.

11 Mori T, Yamazaki R, Aisa Y, Nakazato T, Kudo M, Yashima T *et al.* Brief oral cryotherapy for the prevention of high-dose melphalan-induced stomatitis in allogeneic hematopoietic stem cell transplant recipients. Support Care Cancer, 2006;14(4):392-395.

12 Sato A, Saisho-Hattori T, Koizumi Y, Minegishi M, Iinuma K, Imaizumi M. Prophylaxis of mucosal toxicity by oral propantheline and cryotherapy in children with malignancies undergoing myeloablative chemo-radiotherapy. Tohoku J Exp Med, 2006;210(4):315-320.

13 Ohbayashi Y, Imataki O, Ohnishi H, Iwasaki A, Ogawa T, Inagaki N *et al.* Multivariate analysis of factors influencing oral mucositis in allogeneic hematopoietic stem cell transplantation. Ann Hematol, 2008;87(10):837-845.

14 Bhatt V, Vendrell N, Nau K, Crumb D, Roy V. Implementation of a standardized protocol for prevention and management of oral mucositis in patients undergoing hematopoietic cell transplantation. J Oncol Pharm Pract, 2010;16(3):195-204.

15 Vokurka S, Bystricka E, Scudlova J, Mazur E, Visokaiova M, Vasilieva E *et al.* The risk factors for oral mucositis and the effect of cryotherapy in patients after the BEAM and HD-l-PAM 200 mg/m(2) autologous hematopoietic stem cell transplantation. Eur J Oncol Nurs, 2011;15(5):508-512.

16 Vokurka S, Svoboda T, Jungova A, Karas M, Koza V. Oral cryotherapy can significantly reduce oral mucositis but not acute GVHD incidence in Flu/Mel conditioning allo-SCT. Bone Marrow Transplant, 2012;47(5):739-741.

17 Vokurka S, Chvojkova I, Svoboda T, Brandejsova R, Jungova A, Bystricka E *et al.* The impact of oral cryotherapy and oral and gastrointestinal mucositis after autologous stem cell transplantation. Eur J Oncol Nurs, 2014;18(2):228-229.

18 Batlle M, Morgades M, Vives S, Ferra C, Oriol A, Sancho JM *et al.* Usefulness and safety of oral cryotherapy in the prevention of oral mucositis after conditioning regimens with high-dose melphalan for autologous stem cell transplantation for lymphoma and myeloma. Eur J Haematol, 2014.

19 Svanberg A, Ohrn K, Birgegard G. Five-year follow-up of survival and relapse in patients who received cryotherapy during high-dose chemotherapy for stem cell transplantation shows no safety concerns. Eur J Cancer Care (Engl), 2012;21(6):822-828.
